# *Dientamoeba fragilis* – the most common intestinal protozoan in the Helsinki Metropolitan Area, Finland, 2007 to 2017

**DOI:** 10.2807/1560-7917.ES.2019.24.29.1800546

**Published:** 2019-07-18

**Authors:** Jukka-Pekka Pietilä, Taru Meri, Heli Siikamäki, Elisabet Tyyni, Anne-Marie Kerttula, Laura Pakarinen, T Sakari Jokiranta, Anu Kantele

**Affiliations:** 1 Inflammation Center, Infectious Diseases, Helsinki University Hospital and Helsinki University, Helsinki, Finland; 2 Molecular and Integrative Biosciences Research Programme, Faculty of Biological and Environmental Sciences, University of Helsinki, Helsinki, Finland; 3 Division of Clinical Microbiology, Helsinki University Hospital, HUSLAB, Helsinki, Finland; 4 Medicum, University of Helsinki, Finland; 5 SYNLAB Finland, Helsinki, Finland; 6 Human Microbiome Research Program, Faculty of Medicine, University of Helsinki, Finland

**Keywords:** Dientamoeba fragilis, intestinal, parasite, protozoan, Giardia, dientamoebiasis

## Abstract

**Background:**

Despite the global distribution of the intestinal protozoan *Dientamoeba fragilis,* its clinical picture remains unclear. This results from underdiagnosis: microscopic screening methods either lack sensitivity (wet preparation) or fail to reveal *Dientamoeba* (formalin-fixed sample).

**Aim:**

In a retrospective study setting, we characterised the clinical picture of dientamoebiasis and compared it with giardiasis. In addition, we evaluated an improved approach to formalin-fixed samples for suitability in *Dientamoeba* diagnostics.

**Methods:**

This study comprised four parts: (i) a descriptive part scrutinising rates of *Dientamoeba* findings; (ii) a methodological part analysing an approach to detect *Dientamoeba*-like structures in formalin samples; (iii) a clinical part comparing demographics and symptoms between patients with dientamoebiasis (n = 352) and giardiasis (n = 272), and (iv) a therapeutic part (n = 89 patients) investigating correlation between faecal eradication and clinical improvement.

**Results:**

The rate of *Dientamoeba* findings increased 20-fold after introducing criteria for *Dientamoeba*-like structures in formalin-fixed samples (88.9% sensitivity and 83.3% specificity). A further increase was seen after implementing faecal PCR. Compared with patients with giardiasis, the symptoms in the *Dientamoeba* group lasted longer and more often included abdominal pain, cramping, faecal urgency and loose rather than watery stools. Resolved symptoms correlated with successful faecal eradication (p < 0.001).

**Conclusions:**

Previously underdiagnosed, *Dientamoeba* has become the most frequently recorded pathogenic enteroparasite in Finland. This presumably results from improved diagnostics with either PCR or detection of *Dientamoeba*-like structures in formalin-fixed samples, an approach applicable also in resource-poor settings. Symptoms of dientamoebiasis differ slightly from those of giardiasis; patients with distressing symptoms require treatment.

## Introduction

Despite the worldwide distribution of the enteroparasite *Dientamoeba fragilis*, its prevalence and clinical significance have remained obscure [1,2]. Spread by the faecal-oral route appears most likely [2]. Recently, a cyst stage was discovered [3,4] and its transmission – and association of the protozoan with symptomatic disease – was shown in a rat model [3,4]. Acquisition together with pinworms has also been suggested [1].

Historically, *Dientamoeba* has often remained undetected because of shortcomings of the stool parasite screening methods generally used: formalin-fixed samples were not considered applicable to detecting *Dientamoeba* and wet preparation has poor sensitivity [5]. Instead, diagnosis has required microscopy of specifically stained fresh stool samples (trichrome or modified iron-haematoxylin staining) [6-9] and clinicians familiar with the protozoan knowing how to search for it. Today, PCR methods have become available in many laboratories. The advantages of these methods are obvious, but results from studies using them vary considerably: prevalences between 0.2% and 71% have been reported [2], and some commercial assays have been shown to misidentify the animal protozoan *Tritrichomonas foetus* as *Dientamoeba* [10]. Nevertheless, now that the PCR methods have been widely adopted in the more affluent parts of the world, the reported prevalence of *Dientamoeba* is higher than when the traditional microscopical methods were used [2]. The main reasons for debating the pathogenicity of *Dientamoeba* are its high prevalence in some studies [2] and the large proportion of asymptomatic carriage ranging from 11% [11] to 39% [12].

The most frequently recorded symptoms include abdominal pain and diarrhoea or loose stools [13]; a chronic course lasting up to several years has been reported for 2% [14] to 32% [15] of the patients. Many studies have shown an association between clinical improvement and faecal eradication [2,11,16-19], while the results for children have been conflicting [20-22]. Greater virulence of specific *Dientamoeba* strains has been suggested to account for symptomatic disease. However, recent investigations suggest that there are only two major clonal lines of *Dientamoeba* worldwide, one much more common than the other, which rather points to host characteristics bringing about variation in the clinical picture [23].

In Finland, clinical experience of dientamoebiasis has been accrued since 2007, when our laboratory commenced informing clinicians of *Dientamoeba*-like structures detected in formalin samples, urging them to send in additional trichrome-stained samples. This led to an increase in the number of new findings. Later, in 2017, a multiplex PCR method for protozoan parasites (*Dientamoeba fragilis*, *Giardia lamblia*, *Cryptosporidium parvum*, *Entamoeba histolytica*) was implemented in clinical practice (Amplidiag Stool Parasites test, Mobidiag Ltd, Espoo, Finland) [24]. Spurred by the rise in *Dientamoeba* findings, we conducted a study depicting this development, presenting the formalin sample approach, analyses of demographics and clinical picture, and microbiological and clinical cure rates after antimicrobial therapy.

## Methods

### Study outline

We conducted a retrospective study of the diagnostics and clinical picture of dientamoebiasis. To this end, we collected data on microbiological results and clinical symptoms of patients with a positive finding in clinical faecal samples examined for *Dientamoeba* or *Giardia* in the period from January 2007 to March 2012 at Helsinki University Hospital Laboratory (HUSLAB).

The investigation comprised four parts: (i) a descriptive part relating the annual numbers of new *Dientamoeba* findings from 2007 to 2017; (ii) a methodological part presenting an approach to identify *Dientamoeba* in formalin-fixed samples and comparing the results to those from the same patients’ trichrome samples; (iii) a clinical part comparing the demographics and clinical picture between patients with dientamoebiasis and those with giardiasis; (iv) a therapeutic part analysing faecal eradication and clinical outcome after antiparasitic treatment.

The HUSLAB database was retrospectively searched for *Dientamoeba* and *Giardia* entries dated between January 2007 and March 2012.

The diagnosis of dientamoebiasis was based on positive faecal sample in microscopy after fixation with Ecofix (Meridian Bioscience, Inc., Cincinnati, United States) and modified trichrome staining [25]. In 2017, a multiplex PCR for intestinal protozoa was adopted in routine use and therefore, in the descriptive part of the study, also positive findings by PCR were covered.

Diagnosis of giardiasis was verified by microscopy of formalin-fixed faecal sample, by microscopy of Ecofix-fixed and trichrome-stained faecal sample or by positive antigen test (ProSpecT Giardia/Cryptosporidium Microplate Assay, Oxoid Ltd, Basingstoke, United Kingdom).

Patients with a sample positive for *Dientamoeba* in trichrome staining were included in the *Dientamoeba* group and those with a positive sample in *Giardia* diagnostics comprised the *Giardia* group. To obtain a roughly equal number of patients in both groups, initially only the first five positive results per month were selected for the *Giardia* group. Later, in 2009, when *Dientamoeba* findings exceeded *Giardia* findings, all positive results were recorded in both groups.

### Ethical statement

According to the Finnish Medical Research Act, review by an ethics committee is only required for research involving intervention. The study protocol was approved by the research boards of the Inflammation Center and the regional laboratory of HUSLAB, Helsinki University Hospital (HUH), and the Department of Social Services and Health Care, City of Helsinki.

### Methodological part of the study

Formalin-fixed samples were prepared as follows: faecal samples (2–3 g) were fixed with 10 mL of 10% formalin, filtered through a cheese cloth to remove large debris and concentrated by the standard formalin-ethyl acetate method [26]. Approximately 25 μL of pellet was stained with an equal amount of Lugol’s iodine and examined using bright field microscopy by skilled laboratory personnel at 100× and 400× magnification for 3–5 minutes. In one positive sample, we generally identified several *Dientamoeba*-like structures.

The criteria for *Dientamoeba*-like structures in formalin-fixed samples were as follows: shape slightly flexible, structure round or oval, diameter 8–15 µm; surrounded by a thin cell membrane with no resemblance to *Entamoeba* cysts; coarseness of cytoplasm fine to medium; nuclei (if visible), when stained by iodine, dot-like. The structures looked jumbled because of vacuoles and multiple granules in the cytoplasm.

For photography, Lugol’s iodine solution was added (1:1) and mobility of sample was reduced by adding an equal amount of the fluid and acrylamide solution before polymerisation (40% acrylamide containing 0.5% ammonium persulfate and 0.25% TEMED). The samples were photographed using a 40× objective in a LEICA DM6000 microscope equipped with a LEICA DM2900 camera.

To determine the sensitivity and specificity of detecting *Dientamoeba* in formalin-fixed specimens*,* the results from them were compared with the same patients’ trichrome-stained samples. The samples of both kinds had either been taken at the same time or the trichrome sample shortly afterwards, on the laboratory’s recommendation prompted by findings in the formalin-fixed sample. For the sake of objectivity, we only included in the analyses formalin-fixed samples investigated at least 1 day earlier than the same patient’s trichrome samples (gold standard).

### Clinical part of the study

#### Clinical data

Demographic data, results of faecal microbiological tests and clinical data were retrieved from the electronic patient charts of HUH and Helsinki City healthcare units. The demographic information comprised sex, age and chronic diseases. Symptoms were recorded as reported in patient charts by the clinicians. Data on treatment were collected only for the *Dientamoeba* group.

Additional microbiological data, when available, were collected on faecal pathogens (cultures for *Salmonella* spp., *Yersinia* spp., *Campylobacter* spp. and *Shigella* spp.; culture and toxin test for *Clostridium difficile*; enteric worms and ova (formalin-fixed samples); *Cryptosporidium* spp. (formalin-fixed modified Ziehl-Neelsen staining or antigen test ProSpecT Giardia/Cryptosporidium Microplate Assay, Oxoid Ltd, Basingstoke, United Kingdom); *Entamoeba histolytica* (Entamoeba Celisa Path Test Kit, CeLLabs Pty Ltd, Sydney, Australia); *Enterobius vermicularis* (microscopy of a perianal cotton swab)). Findings of apathogenic faecal microbes, including *Blastocystis hominis,* were recorded separately. Samples screened because of indications other than gastrointestinal symptoms were recorded separately.

Patients with findings positive for other pathogens were excluded. Lack of diagnostic samples for other pathogens did not lead to exclusion since, as opposed to *Dientamoeba* [13], bacterial and viral pathogens are not common as causes of prolonged intestinal complaints [27,28]. The number of patients with missing samples was recorded.

#### Treatment

Our analysis covered the first course of medication, and only patients with at least two control samples taken 2 weeks or more after completing the course were selected. Only data from patients treated with doxycycline, metronidazole, paromomycin or secnidazole – the alternatives recommended in the Finnish guidelines concerning treatment of dientamoebiasis – were included. Data on dosage and regimen duration were recorded when available, yet absence of such data did not result in exclusion because the recommended regimen is uniform in the Finnish guidelines.

Microbiological and clinical outcomes were recorded separately. Faecal clearance was evaluated by findings in trichrome-stained samples. Two or more negative control samples were classified as microbiological success, but even one positive control sample sufficed for interpretation as microbiological failure. Clinical outcome was judged by symptoms recorded in the patient charts before and after antiparasitic treatment. Clinical success was defined as complete or partial relief of symptoms. In our clinical experience, as for giardiasis [29], it is not uncommon for dientamoebiasis symptoms to be partly relieved, with full clinical recovery only occurring weeks after a successful faecal eradication.

### Exclusion criteria

Patients were excluded from the methodological analysis if they had not provided either faecal sample, formalin-fixed or trichrome-stained, or if their trichrome samples had been investigated before their formalin samples. As for the clinical part, patients were excluded because of (i) a verified positive finding of some other intestinal pathogen(s), (ii) missing patient history, (iii) a previously diagnosed active chronic gastrointestinal disease (e.g. colitis ulcerosa) with or without exacerbation or (iv) samples taken as part of routine screening (e.g. recent immigrants). Patients were excluded from post-treatment analysis (i) if they had submitted less than two control samples or the specimens had been taken too early (during the first 2 weeks after completing the treatment), (ii) if they had received some drug other than doxycycline, metronidazole, paromomycin or secnidazole, (iii) if they had been asymptomatic before the treatment, or (iv) if their post-treatment clinical data was missing.

### Statistical analysis

For medians, the range and interquartile range (IQR) were determined. Means with standard deviations and ranges were calculated. For continuous variables, appropriate tests were used (independent samples t-test or Mann–Whitney U-test). For categorical variable analyses, the chi-square test was applied. IBM SPSS Statistics (versions 21 to 24) software was used in statistical analyses.

## Results

### Rates of *Dientamoeba* findings

**Figure 1:**
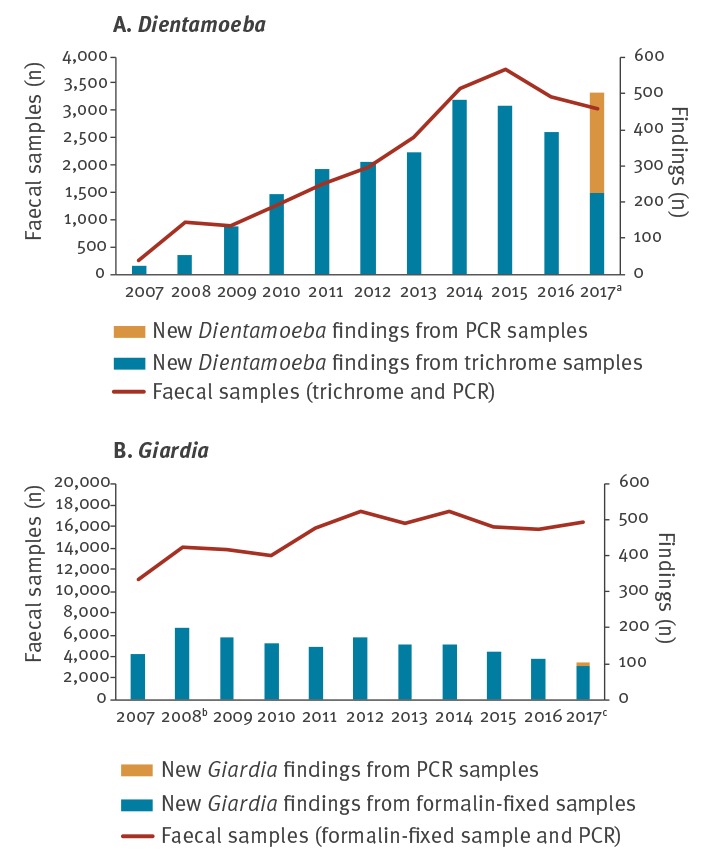
Annual numbers of new *Dientamoeba* and *Giardia* findings and number of faecal samples investigated at HUSLAB, Helsinki, Finland 2007–2017 (n = 189,723 samples)

After adopting the formalin-fixed approach into detection of *Dientamoeba* in 2007, the number of positive trichrome samples multiplied. A steady 20-fold increase in new *Dientamoeba* findings was seen between 2007 and 2017 (Figure 1A), whereas the number of *Giardia* findings remained constant (Figure 1B). Multiplex PCR for *Dientamoeba fragilis*, *Giardia lamblia*, *Cryptosporidium parvum* and *Entamoeba histolytica* was implemented at the beginning of April 2017 and a 28% annual increase in new *Dientamoeba* findings was seen in 2017.

HUSLAB: Helsinki University Hospital laboratory.

^a^ All specimens from 2007–16 were trichrome samples. In 2017, there were a total of 3,065 faecal specimens, consisting of 1,187 PCR and 1,878 trichrome samples.

^b^ The peak in new *Giardia* findings in 2008 was due to a sewage epidemic in the town of Nokia, Finland [40].

^c^ All specimens from 2007–16 were formalin samples. In 2017, there were a total of 16,422 faecal specimens, comprising 15,235 formalin and 1,187 PCR samples.

### Subject groups

During the period from January 2007 to March 2012, we collected a total of 802 *Dientamoeba* and 849 *Giardia* findings from the HUSLAB database (see Methods about equal numbers in the patient groups). Detailed information of these entries was available for 44% (352/802) of the *Dientamoeba* and 32% (272/849) of the *Giardia* patients. Other pathogenic microbes were observed in 6% (20/352) of the *Dientamoeba* and 14% (39/272) of the *Giardia* entries, and apathogenic parasites in 69% (244/352) and 57% (155/272), respectively; a co-infection of *Dientamoeba* and *Giardia* was recorded for 11 patients (Supplementary Table S1).

**Figure 2:**
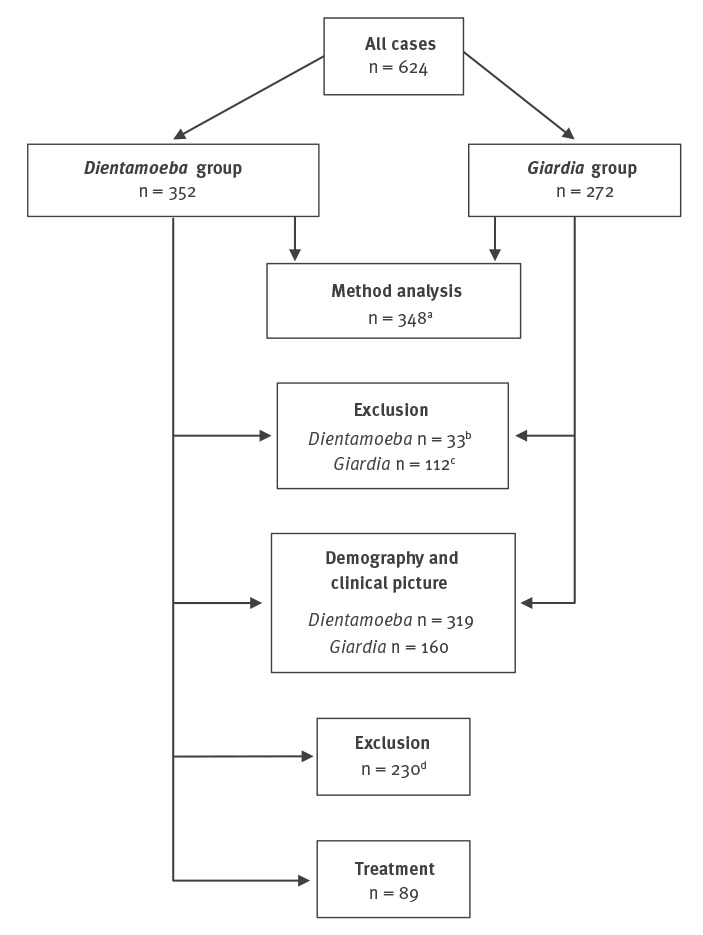
Study design for a retrospective investigation of patients with positive stool findings of *Dientamoeba* and *Giardia*, Helsinki Metropolitan Area, Finland, 2007–2012 (n = 624)

^a^ Patients with both formalin and trichrome samples.

^b^ Exclusion because of: findings of other concomitant pathogens (n = 20), initial diagnosis from immigration screening (n = 6), case history missing (n = 5) or active gastrointestinal disease (n = 2).

^c^ Exclusion because of: findings of other concomitant pathogens (n = 39), initial diagnosis from immigration screening (n = 55), case history missing (n = 17) or active gastrointestinal disease (n = 1).

^d^ Exclusion because of: no data available on treatment (n = 19), less than two faecal control samples collected or control samples taken too early (n = 172), antimicrobial drug other than doxycycline, metronidazole, paromomycin or secnidazole (n = 5), post-treatment clinical data missing (n = 14) or asymptomatic before treatment (n = 20).

After exclusions (Figure 2), the *Dientamoeba* group comprised 319 and the *Giardia* group 160 patients. Apathogenic parasites were identified in the specimens, respectively, of 61% (196/319) and 49% (78/160) of the patients scrutinised, and *Blastocystis hominis* proved the most common apathogen in both groups: 54% (172/319) and 43% (69/160), respectively. Table 1 and Supplementary Table S1 show the number of patients from whom the different faecal pathogens had been analysed and the proportions of positive findings in the microbiological tests among the two final subject groups.

**Table 1:** Number of faecal samples and results of analyses among patients with dientamoebiasis and giardiasis, Helsinki Metropolitan Area, Finland, 2007–2012 (n = 479)

Faecal samples	*Dientamoeba* (n = 319)		*Giardia* (n = 160)	
	n	% of patients	n	% of patients
Formalin-fixed sample^a^				
Patients with sample	289	91	159	99
*Dientamoeba*-positive	251	79	0	0
*Giardia*-positive	0	0	156	98
Trichrome sample				
Patients with sample	319	100	20	13
*Dientamoeba*-positive	319	100	0	0
*Giardia*-positive	0	0	18	11
*Giardia*/*Cryptosporidium* antigen test^b^				
Patients with sample	54	17	37	23
*Giardia*-positive	0	0	25	16
*Cryptosporidium*-positive	0	0	0	0
Enterobiasis cotton swab				
Patients with sample	32	10	13	8
Enterobiasis-positive	6	2	8	5
Faecal bacterial culture				
Patients with sample	166	52	111	69
Sample positive for pathogens^c^	0	0	0	0
*Clostridium* samples^d^				
Patients with sample	68	21	27	17
*Clostridium*-positive	0	0	0	0
*Entamoeba histolytica* sample^e^				
Patients with sample	40	13	10	6
*E. histolytica*-positive	0	0	0	0

^a^ *Dientamoeba* positivity is indicated by *Dientamoeba*-like structures observed in microscopy of formalin sample.

^b^ Formalin-fixed modified Ziehl-Nielsen staining or antigen test (ProSpecT Giardia/Cryptosporidium Microplate Assay, Oxoid Ltd, Basingstoke, United Kingdom).

^c^ Culture for *Salmonella* spp., *Yersinia* spp., *Shigella* spp. and *Campylobacter* spp.

^d^ Culture and toxin test.

^e^ *Entamoeba histolytica* (Entamoeba Celisa Path Test Kit, CeLLabs Pty Ltd, Sydney, Australia).

The demographics were similar for both groups (Table 2): 49% (157/319) of the *Dientamoeba* patients and 54% (86/160) of the *Giardia* patients were female; the age medians were 29 years (IQR: 8–47; range: 1–82 and 31 years (IQR: 20–45; range: 1–76), respectively. The two groups had similar age distributions. An underlying chronic disease was reported for 35% (113/319) of the *Dientamoeba* patients and 32% (51/160) of the *Giardia* patients.

**Table 2:** Demographics of patients with dientamoebiasis and giardiasis, Helsinki Metropolitan Area, Finland, 2007–2012

Characteristics	*Dientamoeba* (n = 319)	*Giardia* (n = 160)	p value
**Sex (n)**			
Male	162	74	0.349^a^
Female	157	86	
**Age (years)**			
Median	29	31	0.266^b^
IQR	8–47	20–45	
Range	1–81	1–76	
**Age groups (n)**			
0–6	64	17	
7–15	66	17	
16–29	32	41	
30–49	85	58	
50–69	61	20	
≥ 70	11	7	
**Chronic diseases (n)**	**113**	**51**	**0.476^a^**

IQR: interquartile range.

^a^ Chi-square test.

^b^ Mann–Whitney U-test.

### *Dientamoeba*-like structures in formalin-fixed samples: specificity and sensitivity

**Figure 3:**
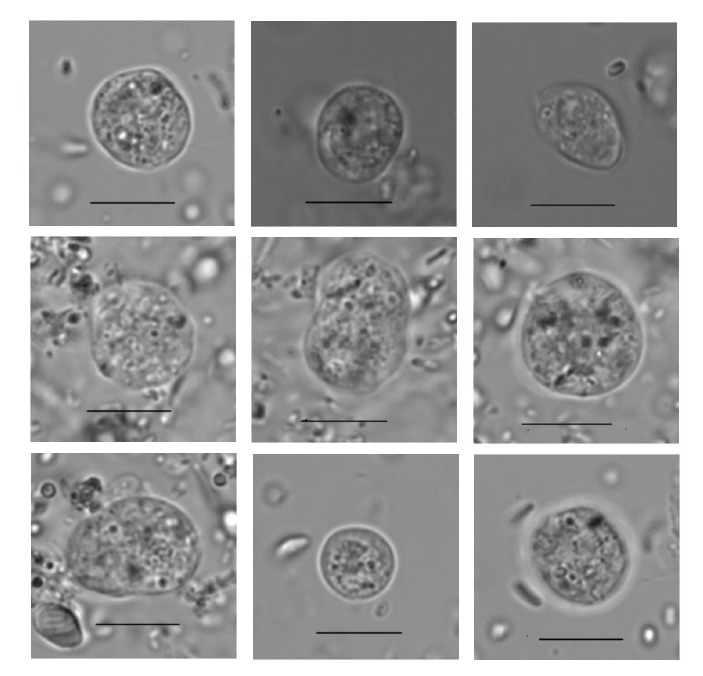
Identification of *Dientamoeba fragilis* from formalin-fixed faecal samples, Helsinki Metropolitan Area, Finland, March 2015 (n = 8)

Figure 3 shows nine representative formalin-fixed samples with *Dientamoeba*-like structures from 8.5 to 17 μm in diameter, round or oval in shape, and with a thin cellular wall. Inside them there were variable numbers (two to six) of darker staining areas with a diameter of ca 0.5 to 1.0 μm. These intracellular structures had a surrounding halo; their identity has not been confirmed, but some of them might be nuclei of the organism. For the formalin sample to be interpreted as positive for *Dientamoeba*-like structures, size and inner structures were the main criteria.

Nine panels showing representative *Dientamoeba*-like structures in formalin samples from eight patients with typical *D. fragilis* trophozoites in trichrome-stained faecal smears. The line indicating the scale in each panel corresponds to 10 µm. The largest *Dientamoeba*-like structures in the picture are seen in the middle panel of the second row and the left panel of the bottom row (13 × 17 and 12 × 17 µm).

A total of 363 patients (320 from the *Dientamoeba* and 43 from the *Giardia* group) had provided both formalin-fixed and trichrome-stained specimens. The trichrome sample had been examined before the formalin sample for 4% (15/363) of patients; these were excluded from further analyses. Judged by results of trichrome staining, 77% (268/348) of the formalin-fixed samples proved true *Dientamoeba* positives, 2% false positives (7/348), 11% false negatives (38/348), and 10% (35/348) true negatives. A sensitivity of 89% (95% confidence interval (CI): 85.2–92.1) and specificity of 83% (95% CI: 68.6–93.3) were recorded for formalin-fixed specimens in detecting *Dientamoeba.*

### Symptoms

A total of 85% *Dientamoeba* and 88% *Giardia* patients had reported symptoms, most commonly diarrhoea, prolonged diarrhoea (lasting over 2 weeks), abdominal pain and flatulence, and abdominal swelling or discomfort (Table 3). The indications for stool sample screening among asymptomatic patients are given in Table 3. *Dientamoeba* patients differed from those with *Giardia* in reporting more frequently loose stools, abdominal pain, constipation and faecal urgency. Symptoms had persisted considerably longer in the *Dientamoeba* group before a correct diagnosis had been made.

**Table 3:** Clinical symptoms of patients with dientamoebiasis and giardiasis, Helsinki Metropolitan Area, Finland, 2007–2012 (n = 479)

Symptoms	*Dientamoeba* (n = 319)		*Giardia* (n = 160)		p value
	n	%	n	%	
Symptomatic	270	85	140	88	NS
Diarrhoea (all)	202	64	116	72	0.034
Watery stools	41	13	54	34	< 0.001
Loose stools	145	46	55	34	0.023
Both watery and loose stools	16	5	7	4	NS
Continuous diarrhoea ≥ 2 weeks	160	50	100	63	0.011
Abdominal pain and cramps	175	55	72	45	0.043
Flatus, abdominal swelling and discomfort	104	33	72	45	NS
Nausea	40	13	46	29	< 0.001
Weight loss	37	12	17	11	NS
Vomiting	30	9	19	12	NS
Fatigue	27	9	12	8	NS
Fever	27	9	24	15	0.025
Constipation	23	7	4	3	0.036
Anal pruritus	23	7	6	4	NS
Faecal urgency	14	4	0	0	0.026
Bloody stools	11	3	2	1	NS^a^
Abnormally smelly stools	8	3	2	1	NS^a^
Heartburn	2	1	2	1	NS^a^
Duration of symptoms before diagnosis (days, median)^b^	180 (IQR: 345; range: 2–3,650)		45 (IQR: 106; range: 1–1,800)		< 0.001^c^

IQR: interquartile range; NS: not significant.

^a^ 2-sided Fisher’s Exact test.

^b^ Data missing for 162 patients in the *Dientamoeba* group and for 97 patients in the *Giardia* group.

^c^ Mann–Whitney U-test.

As for asymptomatic cases: 23 asymptomatic *Dientamoeba* and four asymptomatic *Giardia* cases were found when asymptomatic family members of symptomatic carriers were screened for intestinal parasites. Faecal samples of 18 *Dientamoeba* and 12 *Giardia* asymptomatic cases were examined for unknown reasons. Six *Dientamoeba* and four *Giardia* asymptomatic cases were detected when investigating the subjects’ peripheral venous eosinophilia. Two asymptomatic *Dientamoeba* cases were found in routine examination of faeces from faecal material donors.

Within the *Dientamoeba* group, abdominal pain and cramping had been reported more frequently among the patients younger than 18 years than among the adults (64% vs 49%, p = 0.006, chi-square test); flatulence, abdominal swelling and discomfort had been more commonly listed by the adults (20% vs 42%, p < 0.001, chi-square test).

### Clinical success and parasite eradication

Of the 319 patients with *Dientamoeba*, 28% (89/319) were included in the therapeutic part of the study (Figure 2). Data on dosage and regimen are shown in Supplementary Table S2. The median time until the first control sample was 36 days (IQR: 29–62; range: 16–298) and until the second, it was 76 days (IQR: 40–132; range: 16–378) after completing treatment.

Of all *Dientamoeba* patients treated, clinical success had been reported by the physician for 66% (59/89) and eradication of the parasite from faeces was observed for 53% (47/89). Partial resolution of symptoms was seen among 25 of 59 patients with clinical success. Clinical success was more frequent among patients with successful eradication: 39 of 59 vs eight of 30, with p < 0.001 (chi-square test). The same result was seen in a subgroup analysis of adults (≥ 18 years: n = 63; p < 0.001, chi-square test), whereas in the subgroup of those under 18 years, clinical success did not correlate with eradication of parasites from stool (n = 26; p = 0.683, Fisher’s exact test). When comparing treatment success between the subgroups, faecal clearance was more common among adults than those under 18 years (59% vs 38%), yet the difference was not significant (p = 0.104, chi-square test). Clinical success was not found to differ between the two age groups (68% vs 62%; p = 0.624, chi-square test).

## Discussion

This investigation yielded four noteworthy findings: (i) *Dientamoeba* *fragilis* can be detected in formalin samples with high sensitivity and specificity; (ii) the clinical picture of dientamoebiasis differs in several aspects from that of giardiasis; (iii) faecal clearance of the parasite is associated with alleviation of symptoms especially among the adult population; (iv) in 2017, *Dientamoeba* was the most common pathogenic parasitological finding in the Helsinki Metropolitan Area.

### Formalin fixation approach in diagnostics

While microscopy of wet preparations enables detection of *Dientamoeba* with very low sensitivity [5], formalin-fixed samples have not allowed detection at all. In countries like Finland, where classical formalin-fixed samples have been used as the sole approach (until 2017) to screen for stool parasites, the practise has inevitably led to underdiagnostics of *Dientamoeba*. This is clearly evidenced by the 20-fold upsurge of new *Dientamoeba* findings over the 10-year-period after adopting in 2007 our novel approach to identify *Dientamoeba*-like structures in formalin-fixed samples. Indeed, the number of *Dientamoeba* findings exceeded *Giardia* already before the availability of the PCR methods in 2017. With a specificity of 83.3% and sensitivity of 88.9%, identification of *Dientamoeba*-like structures has proved a viable screening method even if – like all other parasitological microscopy – it requires a trained technician. Nevertheless, as a major advantage over PCR methodology in global settings, by identifying *Dientamoeba* in formalin-fixed samples, diagnostics can also be considerably improved in indigent regions where this is the sole approach available.

### Demographics

Concurring with large epidemiological surveys from Denmark [30], the two largest age cohorts in the *Dientamoeba* group appeared to be daycare and school children, and 30- to 49-year-olds. This finding accords with the conception of *Dientamoeba* infecting especially daycare-aged children and those caring for them [31,32]. This was the first investigation to compare chronic illnesses between patients with dientamoebiasis and those with giardiasis; no significant differences were found.

### Symptoms

As in previous studies [13,15], the *Dientamoeba* group was characterised by a prolonged course of disease (median 180 days). Low awareness of the disease among practitioners and diagnostic difficulties presumably accounted at least partly for such late diagnosis.

The most common symptoms in the *Dientamoeba* group were loose stools, abdominal pain and flatus or abdominal discomfort, all in accordance with earlier studies [2,13]. Our data confirmed previously reported differences in clinical presentations between dientamoebiasis and giardiasis [16]: stomach ache and loose stools were more frequent among *Dientamoeba* patients, while those with *Giardia* more often reported severe illness, watery diarrhoea and even fever. In contrast to a previous investigation [9], faecal urgency was more frequent in the *Dientamoeba* than the *Giardia* group: this may reflect colonic mucosal irritation induced by *Dientamoeba* [2], whereas *Giardia* is generally known to cause disease in the jejunum.

### Treatment success

We evaluated the association between faecal clearance and clinical success. Our data showed a distinct correlation between the two (p < 0.001), consistent with several previous investigations [2,11,16-19]. However, this finding appears to contradict four recent paediatric studies reporting no association between eradication and symptom relief [20,21] or between *Dientamoeba* carriage and symptoms [33,34]. Indeed, scrutinising our data separately for subgroups of children and adults, a difference was revealed: parasitological clearance and clinical outcome were not found to be closely connected (p = 0.683) among children, while a significant correlation was observed (p < 0.001) for adults. This may simply reflect adults’ better skills in describing their symptoms.

The possibility that some of the treatment failures actually were reinfections cannot be ruled out. Transmission within families, especially in households with small children, appears common [35]. Data on family members were not collected, since they are rarely found in patient charts. Variation in failure rates has been shown between various regimens [36], demonstrating that a substantial part of treatment failures are not reinfections.

Doubts about the pathogenicity of *D. fragilis* are presumably related to diagnostic challenges and high proportion of asymptomatic carriers, especially among children [33,34]. In studies with no correlation between clinical and microbiological outcomes, the protozoan has been identified by PCR [20,21], the sensitive method enabling detection of minuscule amounts of microbes [37,38]. No data exist on positive correlation between asymptomatic individuals’ PCR findings and high C_T_ values in PCR. In fact, we found no studies exploring the C_T_ values among symptomatic adult patients without irritable bowel syndrome (IBS); studies applying IBS criteria are not valid for dientamoebiasis, since they only cover patients with a certain selection of symptoms (Rome III criteria). No correlation has been found between dientamoebiasis and IBS-type symptoms in previous investigations [39].

### Limitations

The principal limitations reside in the retrospective study setting and the diagnostics: Firstly, other pathogens were not conclusively excluded from all patients. Viral enteropathogens and diarrhoeagenic *Escherichia coli*, for example, were not tested at HUSLAB during the study period. However, these pathogens typically cause acute watery diarrhoea [28,29], not the clinical picture characteristic of dientamoebiasis that we described. Secondly, in cases where a trichrome sample had not been taken, the exclusion of dientamoebiasis in the *Giardia* group relied on formalin-fixed samples. This should not be a crucial point: presuming a specificity of 83.3% for the formalin-fixed sample to identify *Dientamoeba* would give in the *Giardia* group 23 false-negative results at most. With 23 false-negative *Dientamoeba* patients, the co-infection rate of dientamoebiasis and giardiasis would be 9% (34/375) (Supplementary Table S1). Thirdly, the symptoms could only be reported according to what the clinician had recorded in the patient charts; this limitation applies to the *Dientamoeba* and *Giardia* groups alike. Finally, our study design did not allow estimating the proportion of asymptomatic subjects in the *Dientamoeba* group, which can be assumed to be high.

## Conclusions

We present an increase in the number of *Dientamoeba* findings presumably stemming from implementation of a simple and inexpensive novel diagnostic approach to screen dientamoebiasis from formalin-fixed samples. This approach may prove valuable also – and especially – in low-income countries, where microscopy often remains the only diagnostic laboratory tool available. Already before the implementation of PCR methods, our approach revealed *Dientamoeba* to be much more common in the Helsinki Metropolitan Area than previously thought. In fact, the rate exceeds that of *Giardia*. As abdominal complaints are among the most common reasons for seeking medical care, active efforts are warranted to increase clinicians’ awareness about this pathogen as a cause of prolonged stomach disorders.

## Supplementary Data

56000 Supplement
