# Supplementary material for: Dientamoeba fragilis – the most common intestinal protozoan in the Helsinki Metropolitan Area, Finland, 2007 to 2017
Source: Euro Surveill. 2019 Jul 18;24(29):1800546. doi: 10.2807/1560-7917.ES.2019.24.29.1800546 (PMC6652114; doi:10.2807/1560-7917.ES.2019.24.29.1800546)
Supplement: Supplement [file 1800546_Kantele_Supplement.pdf]

Supplementary material for *Dientamoeba fragilis* – the most common intestinal protozoan in the Helsinki Metropolitan Area, Finland, 2007 to 2017

This supplementary material is hosted by *Eurosurveillance* as supporting information alongside the article [Dientamoeba fragilis – the most common intestinal protozoan in the Helsinki Metropolitan Area, Finland, 2007 to 2017.] on behalf of the authors who remain responsible for the accuracy and appropriateness of the content. The same standards for ethics, copyright, attributions and permissions as for the article apply. *Eurosurveillance* is not responsible for maintenance of any links or email addresses provided therein.

Supplementary Table S1. Other enteric microbes among patients with dientamoebiasis and giardiasis.

Microbes	Dientamoeba (n=352)	Giardia (n=272)
Bacteria		
Yersinia spp.[1]	2	3
Salmonella spp.[1]	1	1
Campylobacter spp.[1]	1	8
Shigella spp.[1]	0	4
Helicobacter[2]	2	4
Pathogenic parasites		
G. lamblia[3]	11	N/A
T. trichura[3]	0	4
A. lumbricoides[3]	1	4
S. stercoralis[3]	0	2
A.duodenale / N. americanus[3]	1	1
T.soliata / solium[3]	1	0
H. nana[3]	0	4
Cryptosporidium spp.[4]	0	4
E. vermicularis[5]	2	2
Schistosoma spp.[6]	0	1
Apathogenic parasites		
B. hominis[3]	190	126
E. nana[3]	77	41
E. coli[3]	39	34
E. dispar / early stages of Entamoeba spp.[3]	31	39
E. hartmanni[3]	15	9
I. butschlii[3]	8	10
C. mesnili[3]	6	7

[1] Bacterial culture samples for *Yersinia, Salmonella, Campylobacter* and *Shigella* missing from 172 patients with *Dientamoeba* and 102 with *Giardia*. [2] *Helicobacter* samples missing from 342 and 257 patients, respectively. [3] Formalin-fixed samples missing from 30 and 1 patients, respectively. [4] Samples (formalin-fixed faecal samples with modified Ziehn-Nielsen staining or antigen test) missing from 247 and 192 patients, respectively. [5] Samples (perianal cotton swab) missing from 318 and 256 patients respectively. [6] Samples (serum antibodies, urine microscopy) missing from all and 270 patients, respectively.

Supplementary Table S2. Medication length and dosing in patients with dientamoebiasis (n=89) treated with doxycycline, metronidazole, paromomycin or secnidazole.

	Doxycycline[1] n=10	Metronidazole[1] n=24	Paromomycin[1] n=35	Secnidazole[1] n=17
Regimen, mean (days)	9.70	9.87	7.00	1.00[2]
Range	3	3	0	0[2]
SD	0.95	0.63	0.00	0.00[2]
Dosage, mean (mg)	100	393.75	474.86	1085.00
Range	0	600	380	1500
SD	0.00	132.13	85.28	644.23
Doses per day	2.0	3.00	2.97	1[2]
Range	0	2	1	0[2]
SD	0.00	0.30	0.17	0.00[2]

[1] Patients with missing data; doxycycline 0, metronidazole 1, paromomycin 0 and secnidazole 7. [2] Secnidazole is given as a single-dose treatment (20mg/kg

Supplementary Table S3. Breakdown of symptoms by various age subgroups among patients with dientamoebiasis.

	Age subgroups											
**Symptom**	**0-6 (n=64)**		**7-15 (n=66)**		**16-29 (n=32)**		**30-49 (n=85)**		**50-69 (n=61)**		**≥ 70 (n=11)**	
Symptomatic	55	86 %	53	80 %	28	88 %	68	80 %	57	93 %	9	82 %
Diarrhoea	45	70 %	35	53 %	23	72 %	50	59 %	44	72 %	5	45 %
Watery stools	6	9 %	8	12 %	2	6 %	13	15 %	10	16 %	2	18 %
Loose stools	25	39 %	25	38 %	18	56 %	35	41 %	28	46 %	2	18 %
Both	2	3 %	2	3 %	3	9 %	2	2 %	6	10 %	1	9 %
Continuous diarrhea ≥ 2 weeks	34	53 %	28	42 %	18	56 %	42	49 %	35	57 %	3	27 %
Abdominal pain and cramps	31	48 %	45	68 %	15	47 %	38	45 %	27	44 %	3	27 %
Flatus, abdominal swelling and discomfort	10	16 %	12	18 %	12	38 %	23	27 %	26	43 %	5	45 %
Nausea	2	3 %	9	14 %	6	19 %	10	12 %	11	18 %	2	18 %
Weight loss	2	3 %	4	6 %	6	19 %	11	13 %	15	25 %	0	0 %
Vomiting	6	9 %	9	14 %	4	13 %	7	8 %	3	5 %	1	9 %
Fatigue	2	3 %	8	12 %	1	3 %	5	6 %	10	16 %	1	9 %
Fever	5	8 %	5	8 %	3	9 %	9	11 %	4	7 %	1	9 %
Constipation	5	8 %	6	9 %	2	6 %	5	6 %	4	7 %	1	9 %
Anal pruritus	7	11 %	3	5 %	2	6 %	6	7 %	5	8 %	0	0 %
Faecal urgency	2	3 %	4	6 %	1	3 %	0	0 %	7	11 %	0	0 %
Bloody stools	3	5 %	0	0 %	1	3 %	3	4 %	4	7 %	0	0 %
Abnormally smelly stools	4	6 %	2	3 %	0	0 %	1	1 %	1	2 %	0	0 %
Heartburn	0	0 %	0	0 %	0	0 %	2	2 %	0	0 %	0	0 %
